# Optimizing Extraction Polarity for Multifunctional Bioactivities and Phenolic Composition in *Polygonum cuspidatum* Sieb. et Zucc. Ultrasonic Extraction

**DOI:** 10.3390/antiox14121431

**Published:** 2025-11-28

**Authors:** Yuchen Cheng, Myat Pwint Phyu, Yuri Kang, Tao Lyu, Woonjung Kim

**Affiliations:** Department of Chemistry, Hannam University, Daejeon 34430, Republic of Koreayuri_1005@nate.com (Y.K.);

**Keywords:** *Polygonum cuspidatum* Sieb. et Zucc., antioxidant, ultrasonic extraction, bioactive compounds

## Abstract

Objective: This study aimed to optimize the extraction of bioactive compounds from *Polygonum cuspidatum* Sieb. et Zucc. (*P. cuspidatum*) by evaluating the effect of ethanol concentration. Methods: Ultrasonic extraction was performed using ethanol concentrations of 0%, 30%, 50%, and 70%, and the resulting extracts were assessed for their chemical composition and multifunctional bioactivities. Results: The 70% Ethanol extract exhibited the highest total polyphenol and flavonoid contents and demonstrated the most potent antioxidant, enzyme-inhibitory, and antimicrobial activities, with significant differences (*p* < 0.05) compared to other concentrations. Chemical analysis identified tannic acid, emodin, and a variety of phenolic compounds as the primary bioactive constituents. Structural analyses using Field Emission Scanning Electron Microscopy (FE-SEM) and Fourier Transform Infrared (FT-IR) Spectroscopy revealed that 70% Ethanol induced the most pronounced structural changes to the cell wall, while FT-IR analysis confirmed the presence of O-H, C=O, C=C, and C-O functional groups, providing a mechanistic basis for the superior extraction efficiency and bioactivity. Conclusions: Ethanol concentration is a critical determinant for maximizing the bioactivity of *P. cuspidatum*. Extraction with 70% ethanol is identified as the optimal condition, supporting the potential of this plant as a source of natural bioactive compounds.

## 1. Introduction

In the context of the rapidly growing global market for functional foods and natural medicines, the development of standardized, high-efficiency ingredients from traditional medicinal plants has become a significant trend [1]. *Polygonum cuspidatum* Sieb. et Zucc., a perennial herb native to East Asia, has been traditionally used to treat inflammation, infections, and circulatory disorders [2,3]. Modern studies have confirmed that its rich polyphenolic content, including stilbenes, anthraquinones, and flavonoids, confers potent antioxidant, antimicrobial, and anti-glycation activities [4,5], making it an ideal candidate for the development of multifunctional natural products. However, translating these bioactivities into stable and efficacious products remains a major challenge, with efficient and controllable extraction of the active constituents being a critical step. The polarity of the extraction solvent is a decisive factor, as it directly influences the selectivity and yield of target compounds [6]. To address this, the present study employed a synergistic strategy, utilizing a tunable, safe, and environmentally friendly ethanol-water system to selectively target bioactive compounds [7], coupled with ultrasonic extraction. Ultrasonic extraction enhances mass transfer and disrupts plant cell structures efficiently at low temperatures through acoustic cavitation, thereby better preserving thermolabile constituents [8,9]. Although the effectiveness of ethanol ultrasonic extraction in extracting bioactive compounds from *P. cuspidatum* has been preliminarily demonstrated [10], a critical bottleneck remains for industrial application. Most previous studies have focused on single bioactivities, leaving the systematic influence of solvent polarity on the phytochemical profile and its relationship to multidimensional bioactivities such as skin health (whitening, anti-wrinkle) and metabolic health (anti-diabetic, anti-obesity) largely unexplored. Moreover, the impact of solvent polarity on the microstructure of plant matrices and its role as a rate-limiting factor in compound release remains unclear, limiting mechanistic guidance for process design. Therefore, this study aims to address this core issue by systematically investigating how the polarity of ethanol-water solvents regulates the chemical composition of *P. cuspidatum* extracts and determines their multifunctional bioactivity profiles. By establishing a comprehensive quantitative link between “solvent composition—plant matrix structural changes—chemical fingerprint—biological function” this work seeks not only to determine the optimal extraction conditions but also to elucidate the underlying mechanism from microstructure to macroscopic efficacy, providing a scientific basis for the value-added and targeted development of *P. cuspidatum* as a renewable source of natural bioactive ingredients and promoting its sustainable application in functional foods, cosmetics, and pharmaceuticals.

## 2. Materials and Methods

### 2.1. Chemicals and Plant Material

The dried rhizomes of *P. cuspidatum* used in this study were purchased from a certified supplier in Yeongcheon, Gyeongsangbuk-do, Republic of Korea, in September 2024. The botanical identity was confirmed by Prof. Woonjung Kim, Professor in the Department of Chemistry at Hannam University, Republic of Korea, and a voucher specimen (Accession No. HNU-PC-2024-0915) has been deposited in the Herbarium of Hannam University, Daejeon, Republic of Korea. The material was fully dried upon receipt and required no additional drying. The rhizomes were ground into a fine powder using a mechanical grinder and subsequently sieved through a 25–40 mesh to obtain a uniform particle size for extraction and analysis. The moisture content of the powdered plant material was determined prior to extraction, and all extraction yields and quantitative analyses are expressed on a dry weight (dw) basis. All reference chemicals, including gallic acid, 1,1-diphenyl-2-picrylhydrazyl (DPPH), 2,4,6-tris(2-pyridyl)-1,3,5-triazine (TPTZ), ABTS diammonium salt, L-3,4-dihydroxyphenylalanine (L-DOPA), mushroom tyrosinase, N-succinyl-(Ala)_3_-p-nitroanilide, porcine pancreatic lipase, and 3-(N-morpholino)propanesulfonic acid (MOPS), were purchased from Sigma-Aldrich (St. Louis, MO, USA). All solvents used for extraction, high-performance liquid chromatography (HPLC), and other analytical procedures were of reagent grade and obtained from Duksan Pure Chemical Co., Ltd. (Seoul, Republic of Korea).

### 2.2. Extraction Procedure

Four distinct dried fractions were prepared from 50 g of *P. cuspidatum* powder by ultrasonic extraction employing ethanol at 0%, 30%, 50%, and 70% (*v*/*v*) with a solvent-to-material ratio of 10:1 (*v*/*w*). After filtration through Whatman No. 2 filter paper and concentration under reduced pressure using a rotary evaporator (WEV-1001 L, Daihan Scientific Co., Ltd., Seoul, Republic of Korea), the extracts were subsequently freeze-dried using a lyophilizer (Model HFD-1, Huachen Instrument Co., Ltd., Zhengzhou, China). The resulting fractions were designated as 0%, 30%, 50%, and 70% ethanol. For subsequent experimental use, all samples were stored at <4 °C and reconstituted in a 70% methanol solution.

### 2.3. Total Polyphenol and Flavonoid Contents in the Ultrasonic Extracts of P. cuspidatum Obtained with Different Ethanol Concentrations

Total polyphenol content was quantified using the Folin–Denis method [11]. Briefly, 12 µL of sample was mixed with 12 µL of Folin–Ciocalteu reagent and allowed to react for 3 min. Then, 180 µL of 10% sodium carbonate was added, and the mixture was incubated in the dark for 60 min. Absorbance was measured at 765 nm using a SpectraMax^®^ ABS Plus microplate reader (Molecular Devices, San Jose, CA, USA). Polyphenol content was calculated from a gallic acid calibration curve and expressed as g GAE/g. All measurements were performed in triplicate.

Total flavonoid content was measured following the method of Jia et al. [12]. A 25 µL aliquot of sample was mixed with 100 µL distilled water and 7.5 µL of 5% sodium nitrite. After 5 min, 15 µL of 10% aluminum chloride was added and allowed to react for 6 min. Then, 50 µL of 1 M sodium hydroxide was added, and absorbance was recorded at 510 nm. Flavonoid content was calculated using a quercetin standard curve and expressed as g QE/g. All measurements were performed in triplicate.

### 2.4. Antioxidant Activities (DPPH, ABTS Radical Scavenging and Ferric Reducing Antioxidant Power (FRAP Assays) in the Ultrasonic Extracts of P. cuspidatum Obtained with Different Ethanol Concentrations

DPPH radical scavenging activity was determined using the method described by Blois [13] and Jeong et al. [14]. A 100 µL sample was combined with 100 µL of 0.2 mM DPPH solution and incubated at room temperature in the dark for 30 min. Absorbance was measured at 517 nm. The blank consisted of 70% ethanol, which was used in place of the sample during the experiment. L-Ascorbic acid served as the reference standard, and IC_50_ values were calculated from the scavenging rate. All measurements were performed in triplicate. The DPPH radical scavenging activity was calculated using the following equation:

$$\begin{array}{c} DPPH\;radical\;scavenging\;activity\;\left(\%\right)\;=\;\left(1-\frac{Absorbance\;of\;the\;sample}{Absorbance\;of\;the\;blank}\right)\times \;100 \end{array}$$

The ABTS radical scavenging activity was generated by reacting ABTS with potassium persulfate for 14–16 h in the dark, as described by Pellegrini et al. [15] and Kim et al. [16]. Before analysis, the solution was diluted with ethanol to obtain an absorbance of 0.70 ± 0.02 at 734 nm. Samples were mixed with ABTS^•^, and absorbance was measured at 734 nm. The blank consisted of 70% ethanol, which was used in place of the sample during the experiment. L-Ascorbic acid was used as the standard for IC_50_ determination. All measurements were performed in triplicate. The ABTS radical scavenging activity was calculated using the following equation:

$$\begin{array}{c} ABTS\;radical\;scavenging\;activity\;\left(\%\right)\;=\;\left(1-\frac{Absorbance\;of\;the\;sample}{Absorbance\;of\;the\;blank}\right)\times \;100 \end{array}$$

FRAP assays were evaluated following Benzie and Strain [17]. The FRAP reagent was prepared by mixing acetate buffer, TPTZ, and FeCl_3_ at a 10:1:1 ratio. A 6 µL sample in 70% methanol was combined with 180 µL of FRAP reagent and 18 µL distilled water, incubated at 37 °C for 10 min, and measured at 593 nm. FRAP values were calculated using an FeSO_4_·6H_2_O standard curve and are expressed as mM/g. All measurements were performed in triplicate.

### 2.5. Tyrosinase and Elastase Inhibitory Activities in the Ultrasonic Extracts of P. cuspidatum Obtained with Different Ethanol Concentrations

Tyrosinase inhibitory activity was measured according to Choi et al. [18]. The reaction mixture contained 125 µL of phosphate buffer (pH 6.8), 50 µL of sample, and 50 µL of 10 mM L-DOPA. The reaction was initiated by adding 25 µL of mushroom tyrosinase (100 U/mL) and incubated at 37 °C for 15 min. Absorbance at 475 nm was recorded, and kojic acid served as the standard. All measurements were performed in triplicate. The inhibitory effect was calculated based on the reduction in dopachrome formation using the following equation:

$$\begin{array}{c} Tyrosinase\;inhibitory\;activity\;\left(\%\right)\;=\;\\ \left(1-\frac{Absorbance\;of\;the\;sample-Absorbance\;of\;the\;blank(buffer\;instead\;of\;enzyme)}{Absorbance\;of\;the\;blank\left(buffer\;instead\;of\;sample\right)}\right)\times \;100 \end{array}$$

Elastase inhibitory activity was determined based on Choi et al. [19]. The assay mixture consisted of 140 µL Tris-HCl buffer (pH 8.0) and 50 µL of N-succinyl-(Ala)_3_-p-nitroanilide. Then, 50 µL of sample and 8 µL elastase solution (pancreatic elastase from porcine pancreas, PPE, 1.4 U/mg) were added, followed by incubation at 37 °C for 20 min. Absorbance was measured at 410 nm. L-Ascorbic acid served as the standard inhibitor. All measurements were performed in triplicate. Elastase inhibitory activity was calculated based on the hydrolysis of the substrate using the following equation:

$$\begin{array}{c} Elastase\;inhibitory\;activity\;\left(\%\right)\;=\\ \left(1-\frac{Absorbance\;of\;the\;sample-Absorbance\;of\;the\;blank(buffer\;instead\;of\;enzyme)}{Absorbance\;of\;the\;blank\left(buffer\;instead\;of\;sample\right)}\right)\times \;100 \end{array}$$

### 2.6. α-Glucosidase and Lipase Inhibitory Activities in the Ultrasonic Extracts of P. cuspidatum Obtained with Different Ethanol Concentrations

The α-glucosidase inhibitory activity was assessed following the method of Eom et al. [20]. A mixture of 90 µL α-glucosidase solution (0.1 U/mL) and 10 µL sample was incubated at 37 °C for 15 min. The reaction was initiated by adding 100 µL of 1 mM p-NPG and further incubated for 5 min. Absorbance was measured at 405 nm. The blank consisted of 70% ethanol, which was used in place of the sample during the experiment. Acarbose served as the standard. All measurements were performed in triplicate. The inhibitory activity was calculated based on the substrate hydrolysis using the following equation:

$$\begin{array}{c} \alpha -Glucosidase\;inhibitory\;activity\;\left(\%\right)\;=\;\left(1-\frac{Absorbance\;of\;the\;sample}{Absorbance\;of\;the\;blank}\right)\;\times 100 \end{array}$$

The lipase inhibitory activity was measured following Kim et al. [21]. Lipase solution was prepared using MOPS, EDTA, and Tris buffer. A 20 µL sample was mixed with the enzyme solution and incubated at 37 °C for 15 min. Then, 4 µL of 10 mM p-NPP was added and incubated for another 15 min. Absorbance at 400 nm was recorded, and orlistat was used as the standard. All measurements were performed in triplicate. The inhibitory activity was calculated based on the hydrolysis of the substrate using the following formula:

$$\begin{array}{c} Lipase\;inhibitory\;activity\left(\%\right)=\\ \left(1-\frac{Absorbance\;of\;the\;sample-Absorbance\;of\;the\;blank(buffer\;instead\;of\;enzyme)}{Absorbance\;of\;the\;blank\left(buffer\;instead\;of\;sample\right)}\right)\times \;100 \end{array}$$

### 2.7. Antimicrobial Activities in the Ultrasonic Extracts of P. cuspidatum Obtained with Different Ethanol Concentrations Against Selected Microbial Strains

The antimicrobial activity of *P. cuspidatum* obtained with different ethanol concentrations was assessed using the disc diffusion method by Bauer et al. [22]. The tested strains, *Staphylococcus aureus* (*S. aureus*) KCTC 1621, *Escherichia coli* (*E. coli*) KCTC 1112, *Pseudomonas aeruginosa* (*P. aeruginosa*) KCTC 2450, and *Candida albicans* (*C. albicans*) KCTC 27,242, were obtained from the Korean Collection for Type Cultures (KCTC) and cultured as summarized in Table 1. After three successive subcultures in nutrient broth at 30 °C or 37 °C for 24–30 h, the suspensions were adjusted to an OD_600_ of 0.2–0.4 (approximately 1 × 10^5^ CFU/mL). The bacterial suspensions were spread onto nutrient agar plates to form uniform lawns. Sterile 8 mm paper discs were loaded with sample solutions at 5 or 10 mg per disc, dried, and placed on the agar surface. Plates were incubated for 24–30 h at the appropriate temperatures, and antimicrobial activity was evaluated by measuring inhibition zone diameters.

### 2.8. Color Characteristics in the Dried Powder of P. cuspidatum Ultrasonic Extracts Obtained with Different Ethanol Concentrations

Color characteristics and surface morphology of *P. cuspidatum* samples treated with 0%, 30%, 50% and 70% Ethanol were analyzed. Sample color was measured in triplicate using a Konica Minolta CR-400 chroma meter (Tokyo, Japan). All measurements were performed under the D65 standard illuminant and a 2° standard observer angle. The L* (lightness), a* (redness), and b* (yellowness) values were recorded, and all data are reported as mean ± standard deviation.

### 2.9. Field Emission Scanning Electron Microscope (FE-SEM) Observation of P. cuspidatum Samples After Extraction with Different Concentrations of Ethanol

The extracted crude powders were mounted onto FE-SEM stubs using conductive double-sided carbon tape. Surface morphology was examined using a JSM-7610F Plus SEM (JEOL, Tokyo, Japan) at Hannam University, with micrographs acquired in secondary electron mode at 5 kV and 1000× magnification.

### 2.10. High-Performance Liquid Chromatography with a Photodiode Array Detector (HPLC-PDA) Analysis in the Ultrasonic Extracts of P. cuspidatum Obtained with Different Ethanol Concentrations

#### 2.10.1. HPLC-PDA Conditions of Tannic Acid and Emodin

Chromatographic conditions for tannic acid and emodin quantification were optimized using a Poroshell 120 EC-C18 column (4.6 × 150 mm, 4 µm; Agilent, Santa Clara, CA, USA) on a YOUNG IN Chromass ChroZen HPLC system with a quaternary pump, autosampler, and PDA detector. Isocratic elution was used for both compounds. Tannic acid was separated with a mobile phase of 50% 0.5% acetic acid in water and 50% methanol for 20 min, with detection at 270 nm. Emodin was separated using 75% 0.1% phosphoric acid in water and 25% methanol for 30 min and detected at 254 nm. For both analyses, the flow rate was set at 1.0 mL/min, the column temperature at 30 °C, and the injection volume at 10 µL.

#### 2.10.2. Method Validation of HPLC-PDA Analysis

Method validation for the HPLC-PDA analysis was performed in accordance with European Commission Decision 2002/657/EC, confirming its linearity, sensitivity, precision, accuracy, and specificity. Linearity was evaluated using standard solutions at multiple concentrations, with calibration curves constructed by plotting peak area (Y) against analyte concentration (X), showing strong correlation coefficients (R^2^) across the tested range. Sensitivity was assessed by determining the limits of detection (LOD) and quantification (LOQ), based on signal-to-noise ratios of 3:1 and 10:1, respectively, and experimentally verified using low-level spiked samples. Precision was evaluated through intra-day and inter-day repeatability, with relative standard deviations (RSD) below 15%. Accuracy and specificity were confirmed using the standard addition method and by comparing chromatograms of blank matrices, standard solutions, and spiked samples, demonstrating reliable quantification and high selectivity of the method.

### 2.11. Fourier-Transform Infrared (FT-IR) Spectroscopy Analysis in the Ultrasonic Extracts of P. cuspidatum Obtained with Different Ethanol Concentrations

Prior to FT-IR examination, the lyophilized ethanol extracts (0%, 30%, 50%, and 70% Ethanol) were pulverized into fine powders using a mortar and pestle. The spectroscopic measurements were performed on a Nicolet™ Summit spectrometer (Thermo Fisher Scientific, Waltham, MA, USA) equipped with an Everest diamond crystal ATR unit and a DTGS KBr detector. Spectral acquisition covered the mid-infrared range from 4000 to 400 cm^−1^ at a resolution of 4 cm^−1^, with each sample undergoing 32 successive scans to improve spectral quality through signal averaging. Background corrections were applied using air as the reference under consistent instrument parameters. Before each analysis, the powdered specimens were evenly distributed on the ATR crystal to ensure optimal surface contact.

### 2.12. Gas Chromatography with a Mass Selective Detector (GC-MSD) Quantification in the Ultrasonic Extracts of P. cuspidatum Obtained with Different Ethanol

Chemical profiling was performed using an Agilent 5977B GC-MSD system (Santa Clara, CA, USA). Samples were injected in splitless mode at 230 °C onto an HP-5ms column (30 m × 0.25 mm, 0.25 µm) with helium as the carrier gas at 1.0 mL/min. The oven was held at 60 °C for 3 min, ramped at 10 °C/min to 300 °C, and maintained for 3 min, giving a total run time of 30 min. After separation, eluents were transferred through a 230 °C transfer line into the mass spectrometer. Mass spectra were recorded in full-scan mode (*m*/*z* 10–600) using electron ionization (EI) under standard instrument settings.

### 2.13. Statistical Analysis in the Ultrasonic Extracts of P. cuspidatum Obtained with Different Ethanol Concentrations

The results from at least three independent experiments are presented as the mean ± SEM. The normality and homogeneity of variances were confirmed using the Shapiro–Wilk and Levene’s tests, respectively. Subsequently, one-way ANOVA with Duncan’s multiple range test was performed using SPSS Statistics 27.0 to assess statistical significance (*p* < 0.05). Figures for GC-MSD and FT-IR spectra were created with SigmaPlot 15.0.

## 3. Results

### 3.1. Extraction Yields

The yields of the extracts increased gradually with increasing ethanol concentrations. The 0%, 30%, 50%, and 70% Ethanol extracts yielded 1.12%, 1.36%, 1.64%, and 2.57%, respectively (Table 2), suggesting that higher ethanol concentrations enhance the extraction efficiency of *P. cuspidatum* constituents. These results indicate that ethanol polarity plays a key role in maximizing the recovery of bioactive compounds from *P. cuspidatum*.

### 3.2. Total Polyphenol and Flavonoid Contents in the Ultrasonic Extracts of P. cuspidatum Obtained with Different Ethanol Concentrations

The total polyphenol and flavonoid contents in *P. cuspidatum* extracts prepared with different ethanol concentrations are summarized in Table 3. Polyphenol quantification, based on a gallic acid standard curve, revealed the highest content in the 70% Ethanol extract (0.74 ± 0.05 g GAE/g), followed by the 50%, 30%, and 0% Ethanol extracts, with values of 0.60 ± 0.06, 0.59 ± 0.04, and 0.48 ± 0.06 g GAE/g. A statistically significant decreasing trend (*p* < 0.05) was observed with reducing ethanol concentration. Similarly, flavonoid content determined using a quercetin standard curve showed the highest level in the 70% Ethanol extract (0.60 ± 0.01 g QE/g), followed by the 50%, 30%, and 0% Ethanol extracts, with values of 0.49 ± 0.07, 0.43 ± 0.06, and 0.24 ± 0.03 g QE/g, also exhibiting a significant concentration-dependent decrease (*p* < 0.05).

### 3.3. Antioxidant Activities (DPPH, ABTS Radical Scavenging and Ferric Reducing Antioxidant Power (FRAP) Assays in the Ultrasonic Extracts of P. cuspidatum Obtained with Different Ethanol Concentrations

The antioxidant activities of *P. cuspidatum* ultrasonic extracts obtained with different ethanol concentrations are summarized in Table 4. In the DPPH radical scavenging activity, L-Ascorbic acid showed the strongest radical scavenging capacity (IC_50_ = 0.004 ± 0.002 mg/mL), followed by the 70%, 50%, 30%, and 0% Ethanol extracts, with IC_50_ values of 0.016 ± 0.001, 0.019 ± 0.001, 0.044 ± 0.000, and 0.050 ± 0.000 mg/mL, respectively. In the ABTS radical scavenging activity, L-Ascorbic acid showed the strongest radical scavenging capacity (IC_50_ = 0.120 ± 0.010 mg/mL), followed by the 70%, 50%, 30%, and 0% Ethanol extracts, with IC_50_ values of 0.237 ± 0.008, 0.262 ± 0.005, 0.319 ± 0.004, and 0.444 ± 0.021 mg/mL, respectively. In the FRAP assay, the 70% Ethanol extract showed the strongest reducing power (1844.370 ± 14.634 mM Fe^2+^/g), followed by the 50%, 30%, and 0% Ethanol extracts, with values of 1790.638 ± 11.234, 1585.760 ± 16.424, and 857.960 ± 12.002 mM Fe^2+^/g, respectively. All assays indicated a statistically significant decreasing trend in antioxidant activity with lower ethanol concentrations (*p* < 0.05).

### 3.4. Tyrosinase and Elastase Inhibitory Activities in the Ultrasonic Extracts of P. cuspidatum Obtained with Different Ethanol Concentrations

Tyrosinase and elastase inhibitory activities of *P. cuspidatum* ultrasonic extracts obtained with different ethanol concentrations are summarized in Table 5. In the tyrosinase inhibitory activity, the 70% Ethanol extract showed the strongest inhibitory capacity (72.60 ± 2.57%), followed by the 50%, 30%, and 0% Ethanol extracts, with inhibition rates of 70.45 ± 3.90%, 68.97 ± 3.21%, and 64.45 ± 2.80%, all of which significantly exceeded the standard Kojic acid (56.31 ± 0.86%). In the elastase inhibitory activity, the 70% Ethanol extract showed the strongest inhibitory capacity (72.30 ± 0.66%), followed by the 50%, 30%, and 0% Ethanol extracts, with inhibition rates of 66.92 ± 4.60%, 62.35 ± 3.70%, and 55.34 ± 3.69%, all of which significantly exceeded the standard L-Ascorbic acid (63.64 ± 0.66%). All assays indicated a statistically significant decreasing trend in inhibitory activity with lower ethanol concentrations (*p* < 0.05).

### 3.5. α-Glucosidase and Lipase Inhibitory Activities in the Ultrasonic Extracts of P. cuspidatum Obtained with Different Ethanol Concentrations

The α-glucosidase and lipase inhibitory activities of *P. cuspidatum* ultrasonic extracts obtained with different ethanol concentrations are summarized in Table 6. In the α-glucosidase inhibitory activity, the 70% Ethanol extract showed the strongest inhibitory capacity (81.04 ± 0.45%), followed by the 50%, 30%, and 0% Ethanol extracts, with inhibition rates of 78.56 ± 1.60%, 76.76 ± 1.10%, and 41.19 ± 0.60%, all of which significantly exceeded the standard Acarbose (34.02 ± 0.38%). In the lipase inhibitory activity, the 70% Ethanol extract showed the strongest inhibitory capacity (84.53 ± 2.79%), followed by the 50%, 30%, and 0% Ethanol extracts, with inhibition rates of 78.32 ± 3.16%, 67.60 ± 1.20%, and 56.16 ± 5.09%, all of which significantly exceeded the standard Orlistat (74.48 ± 1.60%). All assays indicated a statistically significant decreasing trend in inhibitory activity with lower ethanol concentrations (*p* < 0.05).

### 3.6. Antimicrobial Activity in the Ultrasonic Extracts of P. cuspidatum Obtained with Different Ethanol Concentrations Against Selected Microbial Strains

The antimicrobial activities in the *P. cuspidatum* extracts obtained with different ethanol concentrations were assessed by the disc diffusion method at test concentrations of 5.0 mg/disc and 10.0 mg/disc (Table 7 and Table 8). Among all tested extracts, only the 70% Ethanol extract exhibited pronounced and consistent broad-spectrum antimicrobial activity. At 10.0 mg/disc, the inhibition zone diameters against *S. aureus*, *E. coli*, *P. aeruginosa*, and *C. albicans* were 20.00 mm, 9.00 mm, 12.50 mm, and 10.00 mm, respectively. Correspondingly, at 5.0 mg/disc, the inhibition zones measured 10.50 mm, 8.50 mm, 10.50 mm, and 9.00 mm, respectively. Notably, the extracts obtained using 0% Ethanol, 30% Ethanol, 50% Ethanol, 70% Ethanol demonstrated some degree of inhibitory activity against *P. aeruginosa* suggesting that this strain is particularly susceptible to various polar and non-polar phytochemicals in the extracts.

### 3.7. Color Characteristics in the Dried Powder of P. cuspidatum Ultrasonic Extracts Obtained with Different Ethanol Concentrations

The color characteristics of the dried powders obtained from ultrasonic extraction using different ethanol concentrations are summarized in Table 9. As the ethanol concentration increased, the lightness value (L*) increased significantly, reaching a maximum of 55.60 ± 0.01 in the 70% Ethanol extract, indicating a brighter color. Conversely, the redness value (a*) decreased markedly from 6.94 ± 0.04 in the 0% Ethanol extract to 5.27 ± 0.02 in the 70% Ethanol extract. The yellowness value (b*) remained relatively stable in the 0% Ethanol, 30% Ethanol, and 50% Ethanol extracts but showed a significant decline to 24.73 ± 0.65 in the 70% Ethanol extract, indicating a distinct shift in color tone at higher ethanol concentrations.

### 3.8. FE-SEM Observation in the Ultrasonic Extracts of P. cuspidatum Prepared with Varying Ethanol Concentrations

**Figure 1:**
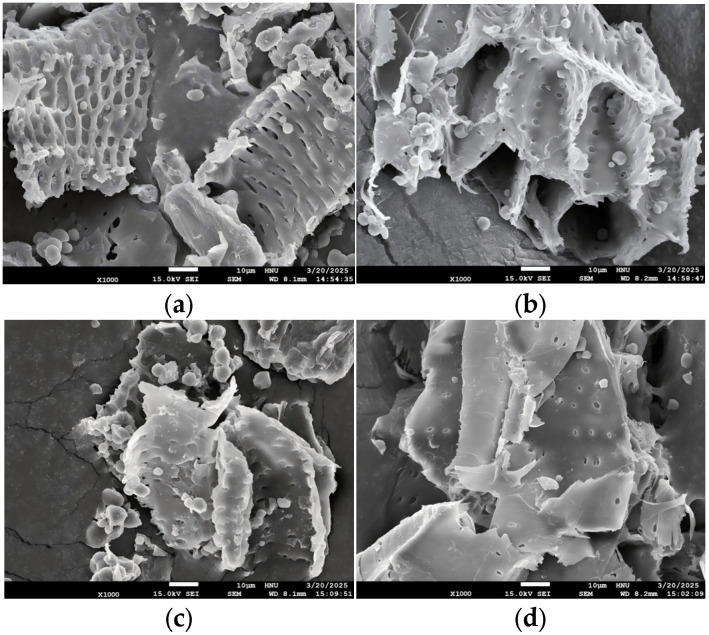
FE-SEM micrographs of the dried powders in the ultrasonic extracts of *P. cuspidatum* in the different ethanol concentrations (magnification: 1000×), (**a**) 0% Ethanol; (**b**) 30% Ethanol; (**c**) 50% Ethanol; (**d**) 70% Ethanol.

The microstructural changes in the *P. cuspidatum* powders in the ultrasonic extracts prepared with varying ethanol concentrations were examined by FE-SEM at 1000× magnification with an accelerating voltage of 5.00 kV. As shown in Figure 1, the sample extracted with 0% Ethanol exhibited a relatively loose surface structure with large and well-preserved pores, despite some degree of surface deformation. With increasing ethanol concentration, the surface morphology became progressively denser, and the pore size significantly decreased. Notably, under the 70% Ethanol condition, the originally porous structure appeared markedly compacted.

### 3.9. HPLC-PDA Quantification of Ultrasonic Extracts of P. cuspidatum Using Different Ethanol Concentrations

**Figure 2:**
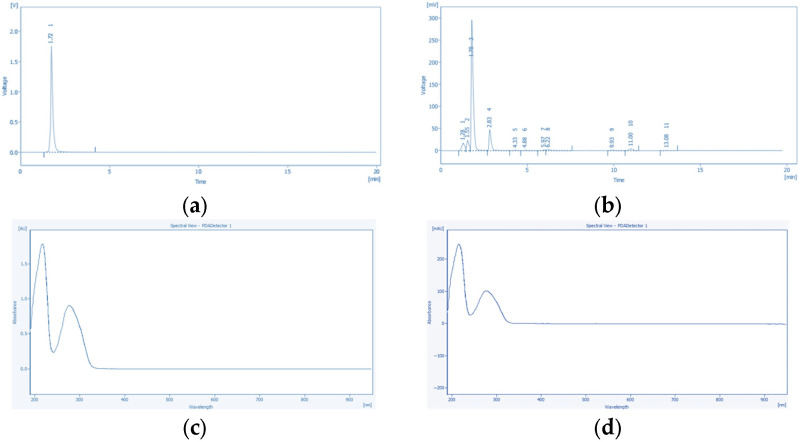
Chromatographic and photodiode array (PDA) data for tannic acid: (**a**) chromatogram of the tannic acid standard; (**b**) representative chromatogram of *P. cuspidatum* extracts prepared with different ethanol concentrations, showing eleven distinct peaks; (**c**) PDA spectrum of the standard; and (**d**) PDA spectrum of the corresponding peak in the sample. The close match between the standard and sample spectra confirms the identification of tannic acid.

**Figure 3:**
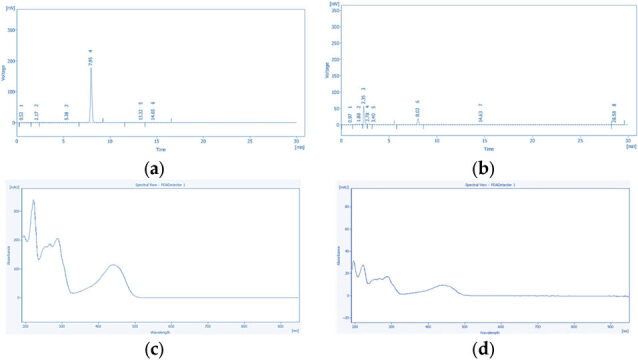
Chromatographic and photodiode array (PDA) data for emodin: (**a**) chromatogram of the emodin standard, showing six peaks; (**b**) representative chromatogram of *P. cuspidatum* extracts prepared with different ethanol concentrations, showing eight distinct peaks; (**c**) PDA spectrum of the standard; and (**d**) PDA spectrum of the corresponding peak in the sample. The close match between the standard and sample spectra confirms the identification of emodin.

Quantification of tannic acid in ultrasonic extracts of *P. cuspidatum* prepared with different ethanol concentrations was performed using HPLC-PDA. Tannic acid showed a retention time of 1.72 min with a symmetrical peak, indicating efficient separation and reliable detection. Emodin was quantified under the same conditions, with a retention time of 7.96 min and well-defined peak shapes, demonstrating consistent method performance. Representative chromatograms and corresponding quantitative results are shown in Figure 2 and Figure 3.

#### 3.9.1. Method Validation of HPLC-PDA Analysis

The HPLC-PDA method showed excellent linearity for both analytes over 25–500 ppm (R^2^ = 0.9970 for tannic acid, 0.9998 for emodin; Table 10). LOD and LOQ were 17.77 and 10.12 ppm for tannic acid, and 1.432 and 3.680 ppm for emodin, confirming reliable low-concentration detection. Precision (RSD) was below 0.01%. Tannic acid eluted at 1.71 min (270 nm) and emodin at 8.01 min (254 nm), both with baseline separation and symmetrical peaks. These results demonstrate the method’s robustness for quantifying tannic acid and emodin in *P. cuspidatum* ultrasonic extracts prepared with different ethanol concentrations.

The precision and accuracy of the HPLC-PDA method were assessed via intra day and inter-day analyses (*n* = 3, Table 11). Tannic acid showed intra-day accuracies of 90.2–98.2% (RSD ≤ 0.15%) and inter-day accuracies of 88.8–104.2% (RSD ≤ 0.14%). Emodin exhibited intra-day accuracies of 93.9–100.7% (RSD ≤ 0.10%) and inter-day accuracies of 94.0–105.9% (RSD ≤ 0.13%). Both compounds exceeded 97% accuracy at 75 ppm, with RSDs ≤ 0.15%, confirming method stability and suitability for quantitative determination of tannic acid and emodin in *P. cuspidatum* ultrasonic extracts prepared with different ethanol concentrations.

#### 3.9.2. Quantification of Tannic Acid and Emodin Using HPLC-PDA

Significant variations in the extraction profiles of tannic acid and emodin were observed in ultrasonic extracts prepared with different ethanol concentrations, as summarized in Table 12 based on HPLC-PDA analysis. For tannic acid, a phenolic compound with amphiphilic characteristics, the highest content was detected in the 70% Ethanol (0.199 ± 0.003 mg/g), followed by 50% Ethanol (0.172 ± 0.005 mg/g) and 30% Ethanol (0.163 ± 0.001 mg/g) extracts, while the lowest content was observed in the 0% Ethanol (0.049 ± 0.001 mg/g), which also exhibited a significant concentration-dependent decrease (*p* < 0.05). For emodin, a low-polarity hydrophobic anthraquinone compound, the highest content was likewise obtained in the 70% Ethanol (0.093 ± 0.011 mg/g), followed by 50% Ethanol (0.053 ± 0.002 mg/g) and 30% Ethanol (0.042 ± 0.004 mg/g) extracts, with the 0% Ethanol showing the lowest content (0.027 ± 0.004 mg/g), which also exhibited a significant concentration-dependent decrease (*p* < 0.05).

### 3.10. FT-IR Spectroscopy Analysis in the Ultrasonic Extracts of P. cuspidatum Obtained with Different Ethanol Concentrations

**Figure 4:**
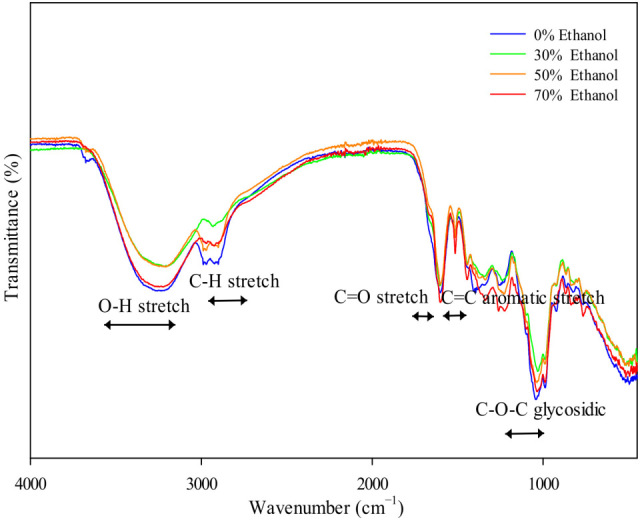
FT-IR Spectroscopy analysis in the ultrasonic extracts of *P. cuspidatum* obtained with different ethanol concentrations.

**Figure 5:**
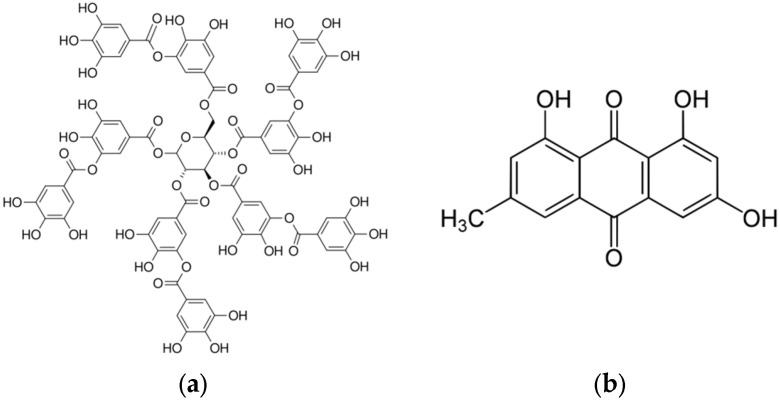
Molecular structures of (**a**) tannic acid, a hydrolyzable tannin composed of a galloyl-glucose complex with multiple phenolic hydroxyl groups, and (**b**) emodin, an anthraquinone derivative featuring a tricyclic aromatic scaffold with hydroxyl and methyl functional groups.

The FT-IR spectroscopy analysis of freeze-dried *P. cuspidatum* extracts prepared by ultrasonic extraction using different ethanol concentrations is shown in Figure 4. illustrating the primary functional groups and characteristic compounds. In the 3550 to 3200 cm^−1^ region, all extracts showed strong and broad absorption bands attributed to O–H stretching vibrations involved in hydrogen bonding, commonly found in phenolics, flavonoids, and carboxylic acids [23,24]. Medium-intensity peaks between 3000 and 2850 cm^−1^ corresponded to aliphatic C–H stretching vibrations, reflecting the presence of methyl (–CH_3_) and methylene (–CH_2_–) groups within fatty acids, terpenoids, and other hydrophobic phytochemicals [25]. Strong absorption peaks observed in the 1700 to 1650 cm^−1^ range were assigned to carbonyl (C=O) stretching vibrations, while peaks in the 1620 to 1500 cm^−1^ range indicated aromatic C=C skeletal vibrations characteristic of aromatic ring structures [26]. Absorption bands from 1300 to 1000 cm^−1^ primarily originated from C–O stretching vibrations of phenolic C–O, esters, ethers, and glycosidic bonds [27]. HPLC-PDA analysis confirmed the presence of tannic acid and emodin (Figure 5) in ultrasonic extracts of *P. cuspidatum* obtained with different ethanol concentrations. Collectively, these results validate the presence of both compounds in the extracts.

### 3.11. GC–MSD Quantification in the Ultrasonic Extracts of P. cuspidatum Obtained with Different Ethanol

**Figure 6:**
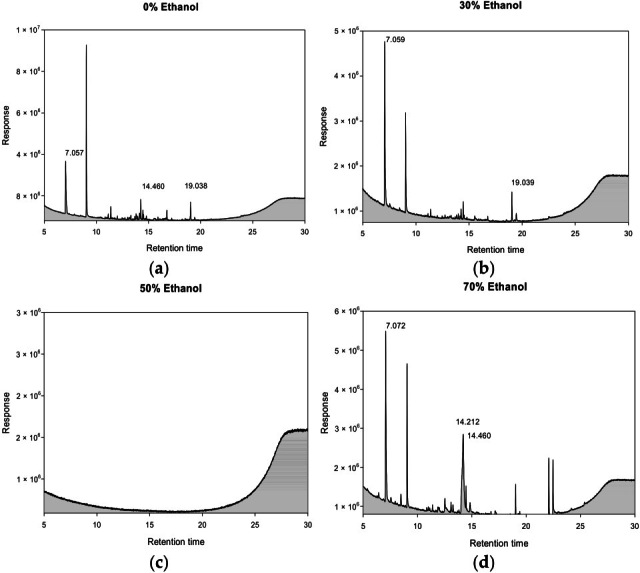
GC–MSD chromatograms in the ultrasonic extracts of *P. cuspidatum* obtained with different ethanol concentrations: (**a**) 0% Ethanol, (**b**) 30% Ethanol, (**c**) 50% Ethanol, and (**d**) 70% Ethanol.

In the quantification of chemical constituents in *P. cuspidatum*, ultrasonic extraction was performed in the presence of different ethanol concentrations, followed by GC–MSD analysis. The extracts obtained with 0%, 30%, 50% and 70% Ethanol were analyzed to reveal the influence of solvent polarity on the composition of secondary metabolites. The 0% Ethanol displayed a distinct chemical profile, primarily composed of polar and moderately polar bioactive compounds. The most abundant constituent was benzhydrazide (30.19%), a compound with documented anti-inflammatory, antioxidant, and antimicrobial properties. Additional notable components included 7,9-di-tert-butyl-1-oxaspiro [4.5]deca-6,9-diene-2,8-dione (6.66%) and 2,4-bis(1,1-dimethylethyl)phenol (2.23%), both contributing to the extract’s antioxidant and antimicrobial activities (Table 13, Figure 6). The 30% Ethanol contained a distinct combination of moderately polar bioactive compounds, with two key constituents identified: 3N′-(4-nitrobenzylidene)benzohydrazide (2.52%) and 1-hydroxy-2-m-nitrophenylimidazole-3-oxide (1.39%) (Table 13, Figure 6). The lower abundance of these compounds compared to the 0% Ethanol extract indicates a shift in chemical profile with increasing ethanol concentration, potentially reflecting differences in solubility and extraction efficiency. The 50% Ethanol revealed no detectable compounds under the current analytical conditions (Table 13, Figure 6). This suggests either: GC-MS analysis of the 70% Ethanol revealed three predominant bioactive compounds (Table 13, Figure 6). The extract was particularly enriched in D-allose (46.58%), a rare sugar derivative with demonstrated antioxidant and anti-inflammatory properties. The second major component, N2-(2-methoxy-5-nitrobenzylideno)benzhydrazide (31.44%), contributed significant antimicrobial activity, while 2,4-bis(1,1-dimethylethyl)phenol (2.28%) added complementary anti-inflammatory and antioxidant effects.

## 4. Discussion

This study systematically evaluated the effect of different ethanol concentrations on the chemical composition and multifunctional bioactivities of *P. cuspidatum* extracts, addressing the knowledge gap regarding the systematic influence of solvent concentration [10]. The results showed that as ethanol concentration increased, the total polyphenol and flavonoid contents also increased, with the highest levels observed in the 70% ethanol extract. This indicates that 70% Ethanol, due to its optimal polarity, can effectively dissolve structurally diverse bioactive compounds and promote plant cell wall disruption, thereby improving the extraction efficiency of phenolic and flavonoid compounds [28,29]. Ultrasonic extraction further enhanced this effect. The cavitation generated by ultrasound produced numerous microbubbles that rapidly collapsed, mechanically disrupting cell walls. Simultaneously, ultrasonic vibration improved solvent penetration and diffusion into plant tissues, reduced the degradation of heat-sensitive components, and preserved the structural integrity and biological activity of the active compounds [8,30,31].

FE-SEM analysis revealed that the surface of *P. cuspidatum* powders became denser and the pore size decreased as ethanol concentration increased. However, the effective contact area increased, providing a physical basis for the efficient release of phenolic and flavonoid compounds [32]. FT-IR, HPLC-PDA, and GC-MSD analyses confirmed the presence of major active constituents, including tannic acid, flavonoids, anthraquinones, and resveratrol derivatives [33,34,35]. FT-IR spectra clearly showed characteristic functional groups, such as O–H, C=O, C=C, and C–O, consistent with literature reports. The 70% Ethanol extract achieved an optimal balance between hydrophilic and hydrophobic compounds, allowing effective solubilization of moderately polar and non-polar compounds such as polyphenols, flavonoids, anthraquinones, and glycosides, thereby enhancing antioxidant, enzyme inhibitory, and antimicrobial activities [36,37,38,39].

Regarding enzyme inhibition, the high activities of α-glucosidase, lipase, tyrosinase, and elastase are likely due to the synergistic action of multiple bioactive compounds. Phenolic and flavonoid compounds can interact with key residues in enzyme active sites via hydrogen bonding, π–π stacking, or metal ion chelation, blocking substrate binding and inducing conformational changes [40,41]. The antimicrobial activity is mainly contributed by tannins and anthraquinones, which can disrupt microbial cell walls and membranes, interfere with enzyme activity, or inhibit DNA replication, leading to growth suppression [42,43]. Differences in ethanol concentration alter the combination of extracted compounds, which may generate synergistic or antagonistic effects, further explaining the variations in multi-target activities [36,44]. Notably, some of the major compounds detected in the extract, such as emodin and tannic acid, have been reported to exhibit antimicrobial activity, suggesting that the extract may contribute to antimicrobial applications [45,46].

Color analysis showed that the 70% Ethanol extract had increased lightness (L*) and decreased redness (a*) and yellowness (b*). This may result from partial degradation or color change in red and yellow anthraquinones during extraction. These changes also correspond to more thorough extraction of intracellular compounds and microstructural alterations [47,48]. High ethanol concentrations reduce solvent surface tension, increase wettability and permeability toward plant tissues, and promote solvent penetration into deep intercellular spaces, thereby enhancing the release of target compounds and improving extraction efficiency and bioactivity [32].

In summary, this study established a direct link between ethanol concentration, plant matrix microstructure, chemical composition, and biological activity. It provides mechanistic insight into the multifunctional bioactivity of *P. cuspidatum* extracts [28,29,30,31,32,44]. The 70% Ethanol extract is the optimal condition for extracting bioactive compounds. The extract is rich in pharmacologically active components and exhibits multi-target bioactivities. This study not only offers mechanistic insight but also provides practical guidance for developing standardized, high-efficiency ingredients from traditional medicinal plants. These findings have potential applications in functional foods, cosmetics, and pharmaceuticals [6,7,10,28,29,30,31,32,33,35,36,39,40,42,43]. A limitation of this study is the lack of systematic toxicological evaluation of the extracts and their major monomeric constituents. Future studies should include in vitro and in vivo safety assessments, combined with mechanistic investigations such as molecular docking, to further clarify the molecular targets and underlying mechanisms of action.

## 5. Conclusions

This study successfully achieved its main objective by systematically demonstrating that solvent polarity (manipulated through ethanol concentration) is a critical determinant of the phytochemical composition and bioactivities of *P. cuspidatum* extracts. The 70% ethanol extract was identified as the most effective, exhibiting the highest phenolic and flavonoid content, which directly correlated with superior antioxidant, enzyme inhibitory, and antimicrobial properties. This confirms that optimizing solvent polarity is essential for unlocking the full functional potential of this plant. Consequently, the 70% ethanol extract of *P. cuspidatum* represents a standardized, multi-functional natural resource with significant promise for applications in functional foods and cosmetics. Future work should focus on the isolation of key bioactive compounds and detailed mechanistic studies to elucidate their synergistic actions.

## Figures and Tables

**Table 1:** Microbial strains and corresponding culture conditions used for antimicrobial activity.

Strain	Growth Media ^1^	Temperature
*S. aureus*	NA/NB	30 °C
*E. coli*	NA/NB	30 °C
*P. aeruginosa*	NA/NB	37 °C
*C. albicans*	NA/NB	30 °C

^1^ NA, Nutrient Agar; NB, Nutrient Broth.

**Table 2:** Yields of the ultrasonic extracts of *P. cuspidatum* obtained with different ethanol concentrations.

Extract	Yields (%, *w*/*w* ^1^)
0% Ethanol	1.12
30% Ethanol	1.36
50% Ethanol	1.64
70% Ethanol	2.57

^1^ The extraction yield (%) was calculated as the ratio of the obtained extract to the dry weight of *P. cuspidatum*.

**Table 3:** Total polyphenol and flavonoid contents in the ultrasonic extracts of *P. cuspidatum* Obtained with Different Ethanol Concentrations.

Extract	Total Polyphenol Content (g GAE/g)	Total Flavonoid Content (g QE/g)
0% Ethanol	0.48 ± 0.06 ^c,1^	0.24 ± 0.03 ^c,1^
30% Ethanol	0.59 ± 0.04 ^b^	0.43 ± 0.06 ^b^
50% Ethanol	0.60 ± 0.06 ^b^	0.49 ± 0.07 ^b^
70% Ethanol	0.74 ± 0.05 ^a^	0.60 ± 0.01 ^a^

GAE: gallic acid equivalent g/g. QE: quercetin equivalent g/g. ^1^ Values are presented as mean ± standard deviation (*n* = 3). ^a–c^ Different superscript letters in the same column indicate statistically significant differences according to Duncan’s test (*p* < 0.05).

**Table 4:** Antioxidant activities (DPPH, ABTS radical scavenging and ferric reducing antioxidant power (FRAP assays) in the ultrasonic extracts of *P. cuspidatum* obtained with different ethanol concentrations.

Extract	DPPH Radical Scavenging Activity IC_50_ (mg/mL) ^1^	ABTS Radical Scavenging Activity IC_50_ (mg/mL) ^1^	FRAP Value (mM Fe^2+^/g)
0% Ethanol	0.050 ± 0.000 ^a,2^	0.444 ± 0.021 ^a,2^	857.960 ± 12.002 ^d,2^
30% Ethanol	0.044 ± 0.000 ^b^	0.319 ± 0.004 ^b^	1585.760 ± 16.424 ^c^
50% Ethanol	0.019 ± 0.001 ^c^	0.262 ± 0.005 ^c^	1790.638 ± 11.234 ^b^
70% Ethanol	0.016 ± 0.001 ^d^	0.237 ± 0.008 ^d^	1844.370 ± 14.634 ^a^
L-Ascorbic acid	0.004 ± 0.002 ^e^	0.120 ± 0.010 ^e^	-

^1^ Inhibitory activity is reported as the mean 50% inhibitory concentration (IC_50_) calculated from three independent measurements by interpolating the concentration–response curve. ^2^ Values are presented as mean ± standard deviation (*n* = 3). ^a–e^ Different superscript letters in the same column indicate statistically significant differences according to Duncan’s test (*p* < 0.05).

**Table 5:** Tyrosinase and elastase inhibitory activities in the ultrasonic extracts of *P. cuspidatum* obtained with different ethanol concentrations.

Extract	Tyrosinase Inhibitory Activity (%) 0.1 mg/mL	Elastase Inhibitory Activity (%) 0.1 mg/mL
0% Ethanol	64.45 ± 2.80 ^b,1^	55.34 ± 3.69 ^c,1^
30% Ethanol	68.97 ± 3.21 ^a,b^	62.35 ± 3.70 ^b,c^
50% Ethanol	70.45 ± 3.90 ^a^	66.92 ± 4.60 ^a,b^
70% Ethanol	72.60 ± 2.57 ^a^	72.30 ± 2.80 ^a^
Standard	56.31 ± 0.86 ^b,2^	63.64 ± 0.66 ^b,3^

^1^ Values are presented as mean ± standard deviation (*n* = 3). ^2^ Kojic acid. ^3^ L-Ascorbic acid. ^a–c^ Different superscript letters in the same column indicate statistically significant differences according to Duncan’s test (*p* < 0.05).

**Table 6:** α-Glucosidase and lipase inhibitory activities in the ultrasonic extracts of *P. cuspidatum* obtained with different ethanol concentrations.

Extract	α-Glucosidase Inhibitory Activity (%) 0.1 mg/mL	Lipase Inhibitory Activity (%) 0.1 mg/mL
0% Ethanol	41.19 ± 0.60 ^d,1^	56.15 ± 5.09 ^d,1^
30% Ethanol	76.76 ± 1.10 ^c^	67.60 ± 1.20 ^c^
50% Ethanol	78.56 ± 1.60 ^b^	78.32 ± 3.16 ^b^
70% Ethanol	81.04 ± 0.45 ^a^	84.53 ± 2.79 ^a^
Standard	34.02 ± 0.38 ^e,2^	74.48 ± 1.60 ^b,3^

^1^ Values are presented as mean ± standard deviation (*n* = 3). ^2^ Acarbose. ^3^ Orlistat. ^a–e^ Different superscript letters in the same column indicate statistically significant differences according to Duncan’s test (*p* < 0.05).

**Table 7:** Inhibition zones in the ultrasonic extracts of *P. cuspidatum* obtained with different ethanol concentrations.

	Extract	Size of Clear Zone (mm)	
		10.0 mg/disc	5.0 mg/disc
*S. aureus*	0% Ethanol	- ^1^	-
	30% Ethanol	-	-
	50% Ethanol	-	-
	70% Ethanol	10.50	20.00
*E. coli*	0% Ethanol	-	-
	30% Ethanol	-	-
	50% Ethanol	-	-
	70% Ethanol	9.00	8.50
*P. aeruginosa*	0% Ethanol	11.00	9.00
	30% Ethanol	11.50	9.50
	50% Ethanol	12.00	10.00
	70% Ethanol	12.50	10.50
*C. albicans*	0% Ethanol	-	-
	30% Ethanol	-	-
	50% Ethanol	-	-
	70% Ethanol	10.00	9.00

^1^ Not detected.

**Table 8:** Photographs showing the antimicrobial effects in the ultrasonic extracts of *P. cuspidatum* obtained with different ethanol concentrations.

Microorganism	0% Ethanol	30% Ethanol	50% Ethanol	70% Ethanol
*S. aureus*	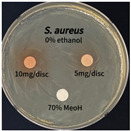	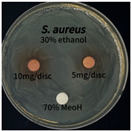	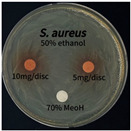	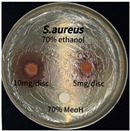
*E. coli*	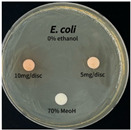	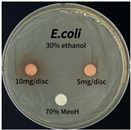	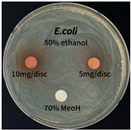	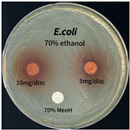
*P. aeruginosa*	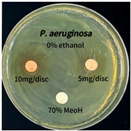	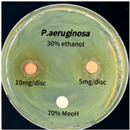	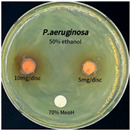	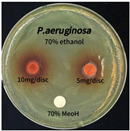
*C. albicans*	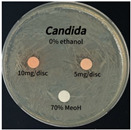	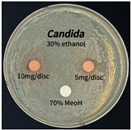	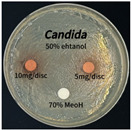	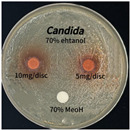

**Table 9:** Color characteristics in the dried powder of *P. cuspidatum* ultrasonic extracts obtained with different ethanol concentrations.

Color Value	0% Ethanol	30% Ethanol	50% Ethanol	70% Ethanol
	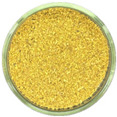	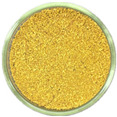	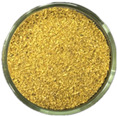	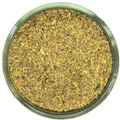
L* (Lightness)	53.04 ± 0.65 ^d,1^	54.67 ± 0.25 ^c^	55.37 ± 0.25 ^b^	55.60 ± 0.01 ^a^
a* (Redness)	6.94 ± 0.04 ^a^	6.41 ± 0.05 ^b^	5.78 ± 0.05 ^c^	5.27 ± 0.02 ^d^
b* (Yellowness)	31.40 ± 0.28 ^a^	31.24 ± 0.33 ^a^	30.40 ± 0.28 ^a^	24.73 ± 0.65 ^b^

^1^ Values are presented as mean ± standard deviation (*n* = 3). ^a–d^ Different superscript letters in the same column indicate statistically significant differences according to Duncan’s test (*p* < 0.05).

**Table 10:** Analytical parameters for tannic acid and emodin in the ultrasonic extracts of *P. cuspidatum* including retention times, linearity, and LOD/LOQ values.

Parameter	Tannic Acid	Emodin
λ ^1^ (nm)	270	254
t_R_ ^2^ (min)	1.71	8.01
RSD ^3^ (%)	0.00	0.01
Linear range (ppm)	25–500	25–500
Regression equation ^4^	Y = 23.53769X − 375.75564	Y = 0.36038X − 1.20597
R ^2,5^	0.9970	0.9998
LODs (ppm)	17.77	1.432
LOQs(ppm)	10.12	3.680

^1^ Wavelength used for detection. ^2^ Retention times. ^3^ Relative standard deviation. ^4^ Calibration parameters with Y representing peak area and X representing analyte concentration (ppm). ^5^ Determination coefficient.

**Table 11:** Precision and accuracy of HPLC-PDA analysis of tannic acid and emodin in ultrasonic extracts of *P. cuspidatum* prepared with different ethanol concentrations.

Compound	Conc. ^1^ (ppm)	Analysis Type	Measured Conc. ^2^ (ppm)	RSD ^3^ (%)	Accuracy ^4^ (%)	RSD (%)
Tannic acid	25	Intra-day (*n* = 3)	24.65	0.05	90.17	0.15
	50		43.89	0.01	98.24	0.02
	75		67.15	0.01	97.36	0.01
	25	Inter-day (*n* = 3)	26.84	0.14	88.78	0.09
	50		47.54	0.07	102.8	0.05
	75		69.39	0.05	104.2	0.07
Emodin	25	Intra-day (*n* = 3)	26.39	0.05	100.7	0.10
	50		50.17	0.05	93.86	0.07
	75		71.92	0.05	100.2	0.06
	25	Inter-day (*n* = 3)	26.84	0.11	94.03	0.13
	50		48.51	0.06	104.0	0.07
	75		70.33	0.09	105.9	0.10

^1^ Nominal concentration of the analyte. ^2^ Determined concentration of the analyte. ^3^ Relative standard deviation. ^4^ Recovery percentage of the analyte.

**Table 12:** Quantification of tannic acid and emodin compounds in the ultrasonic extracts of *P. cuspidatum* obtained with different ethanol concentrations.

Extract	Tannic Acid (mg/g)	Emodin (mg/g)
0% Ethanol	0.049 ± 0.001 ^1,c^	0.027 ± 0.004 ^1,c^
30% Ethanol	0.163 ± 0.001 ^b^	0.042 ± 0.004 ^b^
50% Ethanol	0.172 ± 0.005 ^b^	0.053 ± 0.002 ^b^
70% Ethanol	0.199 ± 0.003 ^a^	0.093 ± 0.011 ^a^

^1^ Values are presented as mean ± standard deviation (*n* = 3). ^a–c^ Different superscript letters in the same column indicate statistically significant differences according to Duncan’s test (*p* < 0.05).

**Table 13:** Chemical constituents identified in the ultrasonic extracts of *P. cuspidatum* obtained with different ethanol concentrations by GC–MSD analysis.

Compound	t_R_^1^ (min)	Chemical Class	Major Compounds (Relative Abundance, %)	Reported Biological Activity
0% Ethanol	7.057	Hydrazone derivative	Benzhydrazide (30.19%)	Anti-inflammatory, Antioxidant, Antimicrobial
	14.46	Phenolic compound	Phenol, 2,4-bis(1,1-dimethylethyl) (2.23%)	Antioxidant, Antimicrobial
	19.04	Spiroketone	7,9-di-tert-butyl-1-oxaspiro [4.5]deca-6,9-diene-2,8-dione (6.66%)	Antioxidant, Antimicrobial
30% Ethanol	7.059	Hydrazone derivative	3N′-(4-Nitrobenzylidene)benzohydrazide (2.52%)	Antimicrobial
	19.04	Imidazole derivative	1-Hydroxy-2-m-nitrophenylimidazole-3-oxide (1.39%)	Antimicrobial
70% Ethanol	7.906	Hydrazone derivative	Benzhydrazide, N2-(2-methoxy-5-nitrobenzylideno) (31.44%)	Antimicrobial
	14.85	Monosaccharide	D-Allose (46.58%)	Antioxidant, Anti-inflammatory
	19.10	Phenolic compound	Phenol, 2,4-bis(1,1-dimethylethyl) (2.28%)	Antimicrobial, Anti-inflammatory, Antioxidant

^1^ Retention time.

## Data Availability

The original contributions presented in this study are included in the article. Further inquiries can be directed to the corresponding author.
